# Prognostic Models for Patient Selection in Palliative Bone Radiotherapy: A Systematic Review

**DOI:** 10.7759/cureus.105473

**Published:** 2026-03-18

**Authors:** Filipa A Martins, Nádia Brito, Domingos Sousa

**Affiliations:** 1 Radiotherapy, Centro Hospital Universitário São João, Porto, PRT; 2 Family Medicine, Unidade de Saúde Familiar Alfa Beja, Unidade Local de Saúde do Baixo Alentejo, Beja, PRT; 3 Health Sciences, Universidade da Beira Interior, Covilhã, PRT; 4 Health Sciences, Instituto Politécnico de Viana do Castelo (IPVC), Viana do Castelo, PRT

**Keywords:** bone metastases, palliative radiotherapy, prognostic models, survival prediction, systematic review

## Abstract

Palliative radiotherapy is a well-established treatment for symptomatic bone metastases, but patients with a vital prognosis of under 3 months may not experience benefit, making accurate prognostic estimation essential for treatment selection. A Preferred Reporting Items for Systematic Reviews and Meta-Analyses (PRISMA)-guided systematic review of PubMed, Scopus, and Web of Science identified studies evaluating prognostic models in patients receiving bone metastases palliative radiotherapy. The risk of bias was assessed using the Prediction model Risk Of Bias ASsessment Tool (PROBAST). Nineteen studies were included, most retrospective and heterogeneous, with mixed primary tumour histology. A broad range of prognostic approaches was identified, including established scores, newly developed prognostic tools, validated models, and individual prognostic factors. Performance status was the strongest prognostic variable. Models found were heterogeneous, and most studies had a high or unclear risk of bias. This PRISMA review highlights that prognostic models may support patient selection for palliative radiotherapy, but given the heterogeneity of patients and clinical settings, their use requires robust external validation and should be adequate to the local context, clinician experience, and patient characteristics.

## Introduction and background

Bone metastases are frequent during the course of advanced malignancies, constituting the third most frequent metastatic site, with an important contribution to worsening quality of life among cancer patients [1,2]. 

Radiotherapy (RT) has long been established as a cornerstone for symptomatic management of painful bone metastases. Another common indication is neurological symptoms due to compression of the spinal cord or nerve roots and postsurgical RT in case of instability [3]. Despite being a very well-tolerated treatment, some side effects are possible: fatigue is reported in a substantial proportion of patients, and a “pain flare” may occur within approximately one week of treatment [4]. Additionally, the extra hospital dislocations, RT positioning, and immobilization are other factors related to additional discomfort for patients with limited life expectancy.

Regarding efficacy, numerous studies report that about 60-70% of patients achieve some degree of pain relief after palliative RT, with complete response in about one-quarter to one-third [3]. Pain relief may begin as early as one week, but an interval of one to four weeks is necessary to experience the maximal pain reduction effect and improvement in quality of life (QoL), depending on the fractionation scheme [3,4]. Consequently, patients with a limited vital prognosis, less than three months, may not experience the symptomatic benefit from RT but still face the burdens of treatment and the discomfort caused by it.

Accurate prognostication is essential in the initial evaluation of patients with bone metastases being considered for palliative RT. The estimation of expected survival is recognized in the European Society for Radiotherapy & Oncology (ESTRO) guidelines for RT in bone metastases as a key factor in treatment selection, dose/fractionation decision, and technique (e.g., conventional palliative vs stereotactic body radiotherapy) [5]. In routine clinical practice, prognostic assessment in patients with bone metastases relies predominantly on performance status, most commonly evaluated using the Karnofsky Performance Status (KPS) and the Eastern Cooperative Oncology Group (ECOG) performance status (PS) scales [6,7].

However, predicting life expectancy in patients with terminal cancer remains challenging in clinical practice. Studies have shown that clinical evaluation overestimates the predicted survival, and patients with very short life expectancy may receive overtreatment in the last two months of their lives [8,9]. Thus, there is a need for objective prognostic models and biomarkers - ideally easy to implement in clinical practice - such as clinical parameters or laboratory data, to assist in selecting patients who are likely to benefit from palliative RT and to avoid futile treatments.

A large US population-based study found that among patients who received radiotherapy in the last 30 days of life, 17.8% underwent more than 10 days of treatment [10].

Given the relevance of prognosis in determining the indication, dose, fractionation, and technique of palliative radiotherapy for bone metastases, this systematic review aims to identify and evaluate prognostic models and biomarkers that may assist in selecting patients eligible for palliative bone metastases RT.

## Review

Materials and methods

This review was performed according to the Preferred Reporting Items for Systematic Reviews and Meta-Analyses (PRISMA) guidelines [11,12]. The review protocol was registered in PROSPERO (CRD420261291478) to ensure methodological rigour and transparency. A comprehensive literature search was performed from inception to 02 March 2026 at PubMed/MEDLINE, Scopus, and Web of Science databases. The search strategy was performed using the MeSH terms (or keywords when not applicable): ‘Bone Metastases’, ‘Radiotherapy, Palliative’, ("Prognostic Model*" OR "Prognostic Score*" OR "Prognostic Factor*" OR "Biomarkers"[Mesh] OR "Survival Prediction" OR "Survival Estimate*") (see Appendices). Furthermore, studies were included if they were conducted in human populations over 18 years of age, including observational or retrospective studies as well as clinical trials. Eligible studies were required to assess biomarkers or prognostic survival scores in patients with bone metastases undergoing palliative or antalgic RT. Studies were excluded if they were narrative or systematic reviews, or if they reported only single case reports. Only Portuguese or English-language papers were included in the search. Unpublished studies or grey literature were not included.

Study selection was performed independently by two reviewers (FAM and DS) who screened titles and abstracts, followed by full-text assessment. Any conflicts in selection were resolved through consultation with a third reviewer (NB).

From the final included studies, data extraction was performed by two reviewers (FAM and DS) using a standardized data extraction form. The following information was extracted from each included study: study characteristics including first author, publication year, and study design; patient characteristics including sample size, type of primary tumor, prognostic factors, outcome,s and validation methods. Any discrepancies in data extraction were resolved through discussion between the two reviewers.

**Figure 1:**
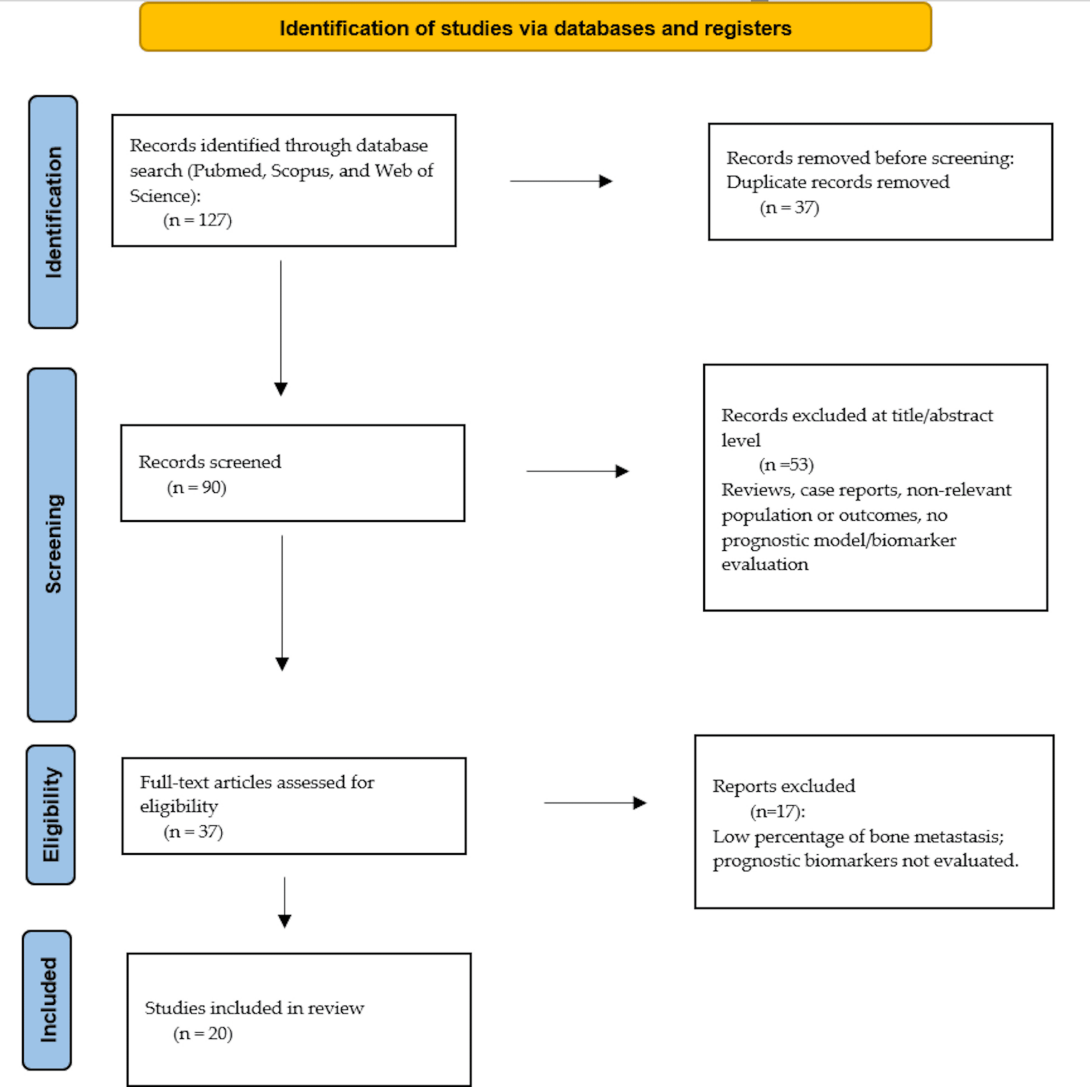
PRISMA 2020 flow diagram for systematic review PRISMA: Preferred Reporting Items for Systematic Reviews and Meta-Analyses

The manuscript was written in accordance with the PRISMA guidelines [11,12]. The risk of bias in the included studies was assessed using the Prediction model Risk Of Bias ASsessment Tool (PROBAST) [13]. A summary of the methodology applied to the database research, according to the PRISMA statement, is presented in Figure 1.

Results

The systematic literature search identified a total of 127 publications. 20 articles were included according to the predefined inclusion and exclusion criteria (Table 1). The studies were published between 2002 and 2026 and predominantly with a retrospective design evaluating clinical and laboratory prognostic factors or established prognostic scores for survival, with insufficient formal internal or external model validation. The majority of the studies initially identify the interest variables using univariable analyses: Cox proportional hazards regression or log-rank tests, and subsequently multivariable Cox regression models to assess their independent association with survival following palliative RT. Sample sizes ranged from 41 to 1157 patients, with large heterogeneity in the study populations (Table 1). Most studies included patients with mixed solid tumors, while a smaller number focused on specific histologies, such as lung cancer, renal cell carcinoma, breast cancer, and melanoma (Table 1).

**Table 1:** Characteristics of included studies Summary of included studies evaluating prognostic models or prognostic factors for patient selection in palliative bone radiotherapy. RT = radiotherapy; SPS = Survival Prediction Score; NRF = number of risk factors; PaP = Palliative Prognostic Score; PAC = palliative appropriateness criteria; ESAS = Edmonton Symptom Assessment System; EQ-5D = EuroQol 5 dimensions; KPS = Karnofsky Performance Status; OS = overall survival; TEACHH = type of cancer, Eastern Cooperative Oncology Group performance status, age, prior palliative chemotherapy, prior hospitalizations, and hepatic metastases; BM: bone metastases; MPT: miscellaneous painful tumor; PPS = Palliative Performance Scale; BMI = Body Mass Index; CCI = Charlson Comorbidity Index

Author (year)	Study design	Primary tumor	Population (n)	Prognostic model/prognostic factors	Model type	Validation method	Outcome predicted
Chow et al. (2002) [14]	Prospective cohort	Mixed solid tumors	Metastatic cancer patients referred for palliative RT (n=395)	SPS: KPS, primary site, metastases site, ESAS symptoms	Prognostic score development (SPS): regression-based	None (apparent performance only)	Median OS: 4.46 months
Chow et al. (2006) [15]	Prospective cohort (validation)	Mixed solid tumors	Spinal bone metastases treated with palliative RT (n=231)	Dutch model; RRRP model	Prognostic score (external validation)	External validation of existing models	Median OS: 7 months
Chow et al. (2008) [16]	Cohort study with development and validation	Mixed solid tumors	Patients referred for palliative RT (training and validation cohorts)	NRF score / simplified SPS	Prognostic score development and validation	Temporal and external validation	NRF = 0 (low risk): 8.8 months; NRF = 1 (intermediate risk): 4.7 months; NRF ≥ 2 (high risk): 1.9 months
Angelo et al. (2014) [17]	Retrospective cohort	Mixed solid tumors	Patients receiving palliative RT (n=412; 579 RT courses)	PRT30 model	Decision-tree prognostic model (recursive partitioning)	Temporal split / independent validation cohort	Median OS: 6.3 months
Bostel et al. (2016) [18]	Retrospective cohort	Melanoma	Patients with spinal metastases from melanoma (n=41)	KPS, neurological status, disease burden	Prognostic-factor analysis	Not applicable (prognostic-factor study)	3-month OS 48.8% 6-month OS 36.6%
Bostel et al. (2019) [19]	Retrospective cohort	Mixed solid tumors	Elderly patients with spinal metastases (n=322)	KPS, neurological status, disease burden	Prognostic-factor analysis	Not applicable (prognostic-factor study)	Median OS: 5.4 months
Howdon et al. (2022) [20]	Secondary analysis of prospective trial	Mixed solid tumors	Patients with bone metastases (n=1,157)	QoL-based prognostic model (EQ-5D)	Prognostic model development and comparison	Internal validation using bootstrap	Median OS: 6.2 months
Makita et al. (2023) [21]	Retrospective cohort	Lung cancer	Lung cancer patients with bone metastases (n=187)	Clinical–laboratory prognostic score	Prognostic score development (regression-based)	None (apparent performance only)	Median OS: 4.3 months
Makita et al. (2024) [22]	Retrospective cohort	Renal cell carcinoma	Renal cell carcinoma patients with bone metastases (n=109)	Clinical prognostic score (derived from regression)	Prognostic score development (regression-based)	None (apparent performance only)	6-month OS 73% 12-month OS 59.4%
Maltoni et al. (2022) [23]	Prospective cohort	Mixed solid tumors	Patients receiving palliative RT (n=376)	PaP, SPS, TEACHH scores	External validation of prognostic score	External validation of PaP, SPS, TEACHH, plus inter-rater analysis	Median OS: 9 months
Nieder et al. (2016) [24]	Retrospective cohort	Breast cancer	Breast cancer patients with bone-only metastases (n=57)	Disease-specific prognostic factors	Prognostic-factor analysis	Not applicable (prognostic-factor study)	Median OS: 23 months
Nieder et al. (2018) [25]	Retrospective cohort	Mixed solid tumors	Patients completing ESAS prior to RT (n=102)	Symptom-based prognostic factors (ESAS)	Prognostic-factor analysis	Not applicable (prognostic-factor study)	Median OS: 6 months
Nieder et al. (2021) [26]	Retrospective cohort	Mixed solid tumors	Patients with bone metastases (n=326)	BMETS score	Machine-learning prognostic model (external validation)	External validation study	Median OS: 7.5 months
Nieder et al. (2023) [27]	Retrospective cohort	Mixed solid tumors	Patients treated with palliative RT (n=219)	PAC score	External validation of prognostic score	External validation study	Median OS: 6 months
Nieder et al. (2023a) [28]	Retrospective cohort	Mixed solid tumors	Elderly patients with bone metastases (n=111)	Rades score	External validation of prognostic score	External validation study	Median OS: 8.2 months
Sakurai et al. (2022) [29]	Retrospective cohort	Mixed solid tumors	Patients with bone metastases (n=376)	NRF; Katagiri scores	Comparative external validation	External validation	Median OS: 7.3 months
Sakurai et al. (2024) [30]	Retrospective validation study	Mixed solid tumors	Patients with painful tumors (n=787)	NRF score	External validation of prognostic score	External validation using independent datasets	Median OS: - BM group: (35.1/10.1/3.3) months; - MPT group: (22.1/9.5/4.6) months
Steinvoort-Draat et al. (2024) [31]	Prospective cohort	Mixed solid tumors	Patients attending rapid response clinic (n=734)	Clinical prognostic factors	Prognostic-factor analysis	Not applicable (prognostic-factor study)	Median OS: 6.4 months
Takeda et al. (2023) [32]	Retrospective cohort	Mixed solid tumors	Patients with vertebral metastases (n=487)	Nomogram (clinical + laboratory variables)	Nomogram-based prognostic model (regression-based)	Internal validation using split-sample	Median OS: 12.4 months
Hennig et al. (2025) [33]	Retrospective cohort	Mixed solid tumors	Patients receiving palliative RT for bone metastases (n=153)	PPS ≥60% vs <60%); RT completion; age; sex, BMI, CCI	Prognostic-factor analysis (Cox regression; PPS-stratified model)	Internal validation	Median OS: 3.6 months

The prognostic methods evaluated across the included studies were heterogeneous and encompassed established prognostic models, externally validated scores, newly developed models, and prognostic-factor analyses. Frequently assessed models included the Survival Prediction Score (SPS), three-variable number-of-risk-factors (NRF) score, Palliative Prognostic Score (PaP), Bone Metastases Ensemble Trees for Survival (BMETS), palliative appropriateness criteria (PAC), Palliative Performance Scale (PPS), and Rades scores, as well as study-specific nomogram-based models and laboratory-based indices (Table 1). The predicted outcomes were primarily overall survival, with one study specifically evaluating 30-day mortality following palliative RT (Table 1).

Validation methods varied markedly across studies. Model validation methods were heterogeneous, including assessment in independent cohorts or internal validation based on resampling or data splitting. However, a substantial proportion of studies reported apparent model performance only, without formal internal or external validation, limiting the interpretability and generalisability of the reported prognostic performance (Table 1).

Risk of Bias Assessment

Risk of bias was assessed for all included studies using the PROBAST, with domain-level and overall judgments summarized in Table 2. The majority of studies were rated as having high or unclear risk of bias, and only a small subset met criteria for low risk of bias across all PROBAST domains (Table 2).

**Table 2:** Risk of bias assessed using the Prediction model Risk Of Bias ASsessment Tool (PROBAST). Each domain was judged as Low, Unclear, or High risk of bias. Low = low risk of bias; High = high risk of bias; Unclear = insufficient reporting to permit a confident judgment, in accordance with PROBAST guidance (i.e., absence of explicit information on participant selection, predictor measurement, handling of missing data, model validation, or performance assessment). Overall risk of bias was judged as High if any domain was rated High; Unclear if one or more domains were Unclear and none were High; and Low only if all domains were rated Low.

Study	Participants	Predictors	Outcome	Analysis	Overall risk of bias
Chow et al. (2002) [14]	Low	Low	Low	High	High
Chow et al. (2006) [15]	Low	Low	Low	Unclear	Unclear
Chow et al. (2008) [16]	Low	Low	Low	High	High
Angelo et al. (2014) [17]	Low	Unclear	Low	High	High
Bostel et al. (2016) [18]	Low	Unclear	Low	High	High
Bostel et al. (2019) [19]	Low	Unclear	Low	High	High
Howdon et al. (2022) [20]	Low	Low	Low	Unclear	Unclear
Makita et al. (2023) [21]	Low	Unclear	Low	High	High
Makita et al. (2024) [22]	Low	Unclear	Low	High	High
Maltoni et al. (2022) [23]	Low	Low	Low	Low	Low
Nieder et al. (2016) [24]	Low	Unclear	Low	High	High
Nieder et al. (2018) [25]	Unclear	Unclear	Low	High	High
Nieder et al. (2021) [26]	Low	Low	Low	Unclear	Unclear
Nieder et al. (2023) [27]	Low	Low	Low	Unclear	Unclear
Nieder et al. (2023a) [28]	Low	Low	Low	Unclear	Unclear
Sakurai et al. (2022) [29]	Low	Unclear	Low	High	High
Sakurai et al. (2024) [30]	Low	Low	Low	Low	Low
Steinvoort-Draat et al. (2024) [31]	Low	Unclear	Low	Unclear	Unclear
Takeda et al. (2023) [32]	High	Unclear	Low	High	High
Hennig et al. (2025) [33]	Low	Low	Outcome	High	High

The Participant’s domain was generally judged to be at low risk of bias, as most studies clearly defined eligibility criteria and study populations (Table 2). Similarly, the Outcome domain was frequently rated as low risk, reflecting the use of objective survival endpoints and appropriate outcome definitions (Table 2). In contrast, the Predictors and Analysis domains frequently showed unclear or high risk of bias. The Analysis domain was the most frequent source of high risk of bias, primarily due to inadequate sample sizes relative to the number of predictors, lack of calibration assessment, and absence of formal model validation in several studies (Table 2).

Discussion

This systematic review identified different prognostic models designed to estimate survival in patients undergoing palliative RT for bone metastases. The included studies were heterogeneous in the included patients, primary tumor histology, and variables incorporated into the model.

*Prognostic Models in Mixed Tumor* *Histology Populations*

Before the work of Chow et al. in 2002, studies evaluating prognostic models were based on small patient series [14]. Chow et al. developed and validated a survival prediction model using a large database from an RT clinic, with 70% of the 395 included patients with bone metastases [14]. Six variables were identified with a significant survival impact: primary tumor histology, location of metastases, KPS, fatigue, appetite, and shortness of breath, assessed using the modified Edmonton Symptom Assessment Scale. Based on these variables, the SPS stratified patients into three prognostic groups with median survivals of 53 weeks, 19 weeks, and eight weeks, with good distinction of survival curves. This model has limitations, particularly in patients with an expected survival of less than three months, which is the relevant cutoff point in selection for palliative RT. The group later evaluated a simplified model, which excluded the patient-reported symptoms, resulting in the three-variable NRF model. This simpler model explained only a limited proportion of survival variability and showed reduced performance compared to the SPS model [16]. In a subsequent study, the SPS model was also compared with a model with only three variables, the Dutch prognostic model. Both models showed excellent calibration between predicted and observed survival (R² = 0.90 and 0.86, respectively), although their scoring systems differed in direction, with higher scores indicating longer survival in the Dutch model and shorter survival in the SPS model. The Dutch model, which includes KPS, primary tumor location, and visceral metastases, is easier to apply in clinical practice [15]. Sakurai et al. performed a secondary analysis of the NRF model in patients with bone and non-bone metastases [29]. Although patients with non-bone metastases had poorer outcomes, the NRF model demonstrated moderate and consistent prognostic accuracy in both groups, with good discrimination power and clear survival separation [29]. Nevertheless, the patient cohort of this study had a better survival than in similar studies, which can compromise generalizing the results. In another work, the NRF model was also compared with the Katagiri scoring system, which incorporates tumor biology, disease burden, ECOG PS, prior treatments, and laboratory parameters. Despite its broader scope, the Katagiri model did not demonstrate superior prognostic accuracy in patients with shorter survival, showing improved performance only in patients with longer prognosis [30].

Howdon et al. evaluated the substitution of clinician-assessed KPS with a patient-reported outcome measure: the EQ-5D self-care dimension. The EQ-5D-based model is comparable to PS in discrimination but did not provide an improvement. The EQ-5D may be particularly interesting in settings without experience in clinical assessment of KPS, being a standardized alternative in this particular context [20].

The Edmonton Symptom Assessment System is a widely used questionnaire in palliative care and addresses 11 major symptoms [34]. Nieder et al. demonstrated that selected patient-reported symptoms may enhance prognostic estimation [25]. This study found that pain at rest and anorexia were the strongest predictors of survival, suggesting that incorporating a limited number of targeted symptom assessments may improve prognostic accuracy while maintaining practicability [25]. However, both EQ-5D and the Edmonton Symptom Assessment System are dependent on patient collaboration, which in a palliative setting can be a limitation, as it is generally accepted that the burden of questionnaires should be minimized for patients near the end of life.

The BMETS model is a clinical decision support tool with 27 prognostic variables, including KPS, recent systemic therapy, and haematological parameters. BMETS correctly predicts median survival in 68% of patients with less than three months of survival [26]. Considerable deviations were observed in a subset of patients, predicting longer survival for patients who died within three months of starting RT. International practice variations and the time required for data collection are important model’s limitations.

Angelo et al. developed, to our knowledge, the only prognostic model designed to identify patients at high risk of death within 30 days after palliative RT [17]. The model incorporated six variables: lung or bladder cancer, ECOG PS 3-4, low haemoglobin, opioid analgesic use, steroid use, and known progressive disease outside the RT target volume, and achieved a correct identification rate of 75%. Despite methodological limitations, this study highlighted the persistent use of palliative RT in the final month of life, underscoring the difficulty clinicians face in limiting interventions even when prognosis is recognized as poor.

Takeda et al. proposed a six-factor prognostic score for patients with vertebral metastases incorporating clinical and haematological variables, including history of chemotherapy, primary tumor, analgesic use, neutrophil-to-lymphocyte ratio, serum albumin, and lactate dehydrogenase [32]. Median survival differed significantly across the four risk groups, indicating good prognostic discrimination. Despite being a theoretically complete model, in clinical practice, it isn’t easy to implement routinely. The authors noted limitations related to missing ECOG PS data, incomplete laboratory results, and incomplete pathological tumor information.

Maltoni et al. prospectively evaluated the accuracy of the PaP score, and also made a very interesting analysis of the interrater variability of PaP, SPS, and TEACHH scores [23]. PaP score includes six variables (KPS, clinical prediction of survival, anorexia, dyspnoea, total white blood count, and lymphocyte percentage). The accuracy of the PaP score for predicting 30-day survival was 74.8% for radiation oncologists and 80.7% for palliative care physicians. Interrater variability for the PaP score was slightly higher than for more objective scores. The TEACHH model showed an accuracy of 76.1% for radiation oncologists and 64.7% for palliative care physicians in predicting survival.

Hennig et al. recently evaluated the prognostic value of the PPS in a retrospective cohort of 153 patients receiving palliative RT for bone metastases [33]. The study demonstrated that a PPS ≥60% was independently associated with longer overall survival, with a median survival of 3.6 months for the overall cohort. In multivariable analysis adjusting for age, sex, comorbidity burden, and treatment-related variables, PPS remained a significant predictor of survival. RT completion emerged as the strongest prognostic factor; however, this likely reflects selection bias, as patients with better baseline functional status were more likely to complete treatment. Unlike some prognostic models, PPS was not associated with discharge destination, highlighting the influence of non-clinical factors in post-treatment care pathways. Although limited by its retrospective design and lack of external validation, this study reinforces the central role of PS as a simple and clinically applicable determinant of survival in patients undergoing palliative bone RT.

Pathology-Specific Prognostic Models

Most studies evaluate patients with bone metastases from a miscellaneous primary tumor, with a high proportion of breast and prostate cancer representation. Pathology-specific studies allow a more precise evaluation of tumor-specific prognostic factors, particularly in malignancies associated with poorer outcomes [5]. Bostel et al. evaluated a small patient group with spinal metastases from malignant melanoma and identified KPS ≥70, multiple bone metastases, and concomitant visceral metastases as significant predictors of poor survival. Median survival was less than 14 months, with only 37% of patients alive six months after RT. Despite methodological limitations, this study is, to our knowledge, the only work on melanoma patients [18]. It is especially relevant since the number of patients requiring palliative RT is likely to increase with the advent of new targeted therapies. Similarly, Makita et al. developed prognostic scoring systems for lung and renal cancer patients, confirming the prognostic relevance of ECOG PS, visceral metastases, molecular-targeted therapies, and disease control [21,22]. Nieder et al. analyzed prognostic factors in a small cohort of breast cancer patients, identifying hormonal receptor status, the lactate dehydrogenase and alkaline phosphatase levels as prognostic markers. Superior prescription dose appears as a significant prognostic factor, being a bias of the study, since the physician can identify patients with a favourable prognosis and treat them with higher doses [24]. It is important to conduct prostate and breast-specific studies. The general prognostic models may be insufficiently sensitive to identify patients with shorter survival in these frequently indolent subtypes. Notably, the only existing study on breast cancer included a very small number of patients and presented the limitations already discussed.

Geriatric Patients

The number of elderly patients with cancer is increasing. This population is particularly challenging due to increased comorbidity, higher treatment-related toxicity, and shorter expected survival. In the two studies focusing on elderly patients, performance status was identified as the strongest predictor of survival, independent of chronological age, although prognostic group separation was less distinct in some models [19,28].

The studies included in this review highlight that PS consistently emerged as the most influential prognostic factor across diverse models. However, its subjective assessment and potential for rapid deterioration limit its reliability. The use of laboratory parameters provides a more complete assessment of patient status; for example, low albumin levels, elevated LDH, or neutrophil-to-lymphocyte ratio reflect nutritional status and systemic inflammation. However, such data are not always available in palliative settings, and prognostic assessment should not require additional invasive procedures. The past history of chemotherapy is also a valuable piece of information, since it can be indicative of disease burden. Analgesic use, particularly the use of opioids, is associated with more extensive and aggressive bone disease and has been linked to poorer survival.

Studies such as Maltoni et al. demonstrate that a balance between prognostic accuracy and clinical feasibility remains a central challenge [23]. More complex models may offer greater prognosis discrimination, but are often limited by data availability, time constraints, and variability in clinical practice. Importantly, clinician experience influences prognostic estimation, and selecting a model appropriate to both the patient population and clinical context may be more important than score discrimination power.

Future prognostic models may benefit from incorporating tumor biology, circulating biomarkers, and artificial intelligence-based tools to optimize and support clinical decisions.

This systematic review has several limitations. First, although a comprehensive search strategy was employed, it is possible that relevant studies were missed, particularly unpublished models or conference abstracts, which were excluded by design. Second, the majority of included studies were retrospective and single-center in nature, with substantial heterogeneity in patient populations, primary tumor types, prognostic variables, and outcome definitions, precluding quantitative synthesis or meta-analysis. Third, reporting quality was variable, with frequent insufficient detail regarding predictor measurement, handling of missing data, and model development procedures, which limited the assessment of risk of bias using PROBAST. In addition, few studies performed formal external validation, and most models were developed or tested in highly selected populations, limiting generalisability. Finally, survival outcomes were inconsistently reported across studies, with some reporting stratified survival or survival rates rather than median post-RT survival, further complicating direct comparison between models.

## Conclusions

The prognostic models and biomarkers identified in this systematic review show potential to support decision-making in palliative bone RT. However, robust external validation and prospective multicenter studies are required before their integration into clinical algorithms. Given the heterogeneity of patients, healthcare settings, and available resources, the choice of prognostic score should be individualized, with each clinical team adopting the model best suited to its specific context, clinician expertise, and patient population.

## Appendices

Search query

PUBMED

Equação de pesquisa: ("Bone Metastases"[Mesh] OR "Bone Metastasis") AND ("Radiotherapy, Palliative"[Mesh] OR "Palliative Radiotherapy" OR "Antalgic Radiotherapy") AND ("Prognostic Model*" OR "Prognostic Score*" OR "Prognostic Factor*" OR "Biomarkers"[Mesh] OR "Survival Prediction" OR "Survival Estimate*")

Scopus

TITLE-ABS-KEY("Bone Metastases" OR "Bone Metastasis") AND TITLE-ABS-KEY("Palliative Radiotherapy" OR "Antalgic Radiotherapy" OR "Radiotherapy, Palliative") AND TITLE-ABS-KEY("Prognostic Model*" OR "Prognostic Score*" OR "Prognostic Factor*" OR "Biomarker*" OR "Survival Prediction" OR "Survival Estimate*")

Web of Science

Equação de pesquisa: ("Bone Metastases"[Mesh] OR "Bone Metastasis") AND ("Radiotherapy, Palliative"[Mesh] OR "Palliative Radiotherapy" OR "Antalgic Radiotherapy") AND ("Prognostic Model*" OR "Prognostic Score*" OR "Prognostic Factor*" OR "Biomarkers"[Mesh] OR "Survival Prediction" OR "Survival Estimate*")
